# ERG Responses in Albinism, Idiopathic Infantile Nystagmus, and Controls

**DOI:** 10.1167/iovs.65.4.11

**Published:** 2024-04-04

**Authors:** Zhanhan Tu, Christopher Degg, Michael Bach, Rebecca McLean, Viral Sheth, Mervyn G. Thomas, Shangqing Yang, Irene Gottlob, Frank A. Proudlock

**Affiliations:** 1 University of Leicester Ulverscroft Eye Unit, School of Psychology and Vision Sciences, University of Leicester, Robert Kilpatrick Clinical Sciences Building, Leicester Royal Infirmary, Leicester, United Kingdom; 2 Medical Physics and Clinical Engineering, Nottingham University Hospitals NHS Trust, United Kingdom; 3 Eye Center, Freiburg University, Killianstraße 5, Freiburg, Germany; 4 Gonville and Caius College, University of Cambridge, Cambridge, United Kingdom; 5 Cooper University Hospital, Camden, United States

**Keywords:** albinism, electroretinography, nystagmus, infantile nystagmus

## Abstract

**Purpose:**

Our primary aim was to compare adult full-field ERG (ffERG) responses in albinism, idiopathic infantile nystagmus (IIN), and controls. A secondary aim was to investigate the effect of within-subject changes in nystagmus eye movements on ffERG responses.

**Methods:**

Dilated Ganzfeld flash ffERG responses were recorded using DTL electrodes under conditions of dark (standard and dim flash) and light adaptation in 68 participants with albinism, 43 with IIN, and 24 controls. For the primary aim, the effect of group and age on ffERG responses was investigated. For the secondary aim, null region characteristics were determined using eye movements recorded prior to ffERG recordings. ffERG responses were recorded near and away from the null regions of 18 participants also measuring the success rate of recordings.

**Results:**

For the primary aim, age-adjusted photopic a- and b-wave amplitudes were consistently smaller in IIN compared with controls (*P* < 0.0001), with responses in both groups decreasing with age. In contrast, photopic a-wave amplitudes increased with age in albinism (*P* = 0.0035). For the secondary aim, more intense nystagmus significantly reduced the success rate of measurable responses. Within-subject changes in nystagmus intensity generated small, borderline significant differences in photopic b-wave peak times and a-and b-wave amplitudes under scotopic conditions with standard flash.

**Conclusions:**

Age-adjusted photopic ffERG responses are significantly reduced in IIN adding to the growing body of evidence of retinal abnormalities in IIN. Differences between photopic responses in albinism and controls depend on age. Success at obtaining ffERG responses could be improved by recording responses at the null region.

Full-field ERG (ffERG) is a technique used to detect abnormal responses to photopic and/or scotopic light stimulation. It is commonly used in infantile nystagmus (IN), an involuntary oscillation of the eyes occurring in approximately 1.4 per 1000 individuals,^1^ because IN is often associated with retinal deficits. Severe morphological abnormalities of the fovea, optic nerve head and peripapillary retinal nerve fiber layer have been observed in albinism using optical coherence tomography (OCT).^2^^–^^7^ Previously reported ffERG responses in albinism are equivocal with some literature reporting normal ERG responses, and others reporting abnormal responses.^8^^–^^13^

Structural changes of the retina and optic nerve head have also recently been demonstrated for idiopathic IN (IIN) using OCT, which has historically been described as not being associated with afferent visual deficits. People with mutations in the *FRMD7* gene, a common form of IIN, have significantly shallower foveal pits, decreased optic disk and cup areas, reduced cup depths, and thinner retinal nerve fiber layers.^14^ ffERG responses are reported to be normal in IIN.^8^^–^^10^ Recent studies in mice have found that the FRMD7 protein is expressed specifically in starburst amacrine cells and *FRMD7* mutations lead to abnormalities in cellular responses.^15^ Amacrine, bipolar, and/or ganglion cells have been suggested to be involved in the generation of oscillatory potentials (OPs).^16^^–^^18^ OPs are measured in standard ffERG testing in humans; however, they have not been reported previously for albinism and IIN.

Given the inconsistency in ERG responses reported in the literature and difficulties in controlling for eye movement artifacts caused by nystagmus, we have revisited the question of ffERG responses in albinism and IIN compared with controls. Limitations of the methodology used in previous studies of ERG responses in albinism and IIN include (i) often small sample sizes, (ii) mostly use of skin electrodes with a relatively lower signal-to-noise ratio,^19^^,^^20^ (iii) studies usually performed without pupil dilation, (iv) short dark adaptation times, which may cause changes in the sensitivity of photoreceptors, which may not totally recover in light pigmented participants, and (v) all previous human studies use children who are less cooperative than adults.

The primary aim of this study was to record ffERG responses in a large group of adult participants, with albinism (*n* = 68) and IIN (*n* = 43) in comparison with controls (*n* = 24) using DTL electrodes, which contact the corneal margin. We also compare the ratio of cone/rod responses and OPs between groups. Our secondary aim was to investigate the effect of artefacts caused by nystagmus-related eye movements on ffERG responses by making within subject comparisons of ffERG responses when nystagmus is less intense (i.e., at the null region) to when it was more intense nystagmus (i.e., away from the null region) in participants with a range of retinal deficits. We achieved this by selecting participants with marked null regions and recording ffERG responses as they adopted head postures. A marked null region was defined as at least a two-fold increase in nystagmus intensity when participants held gaze away from their null region.

## Methods

### Ethics, Consent, and Recruitment

This study follows the tenets of the Declaration of Helsinki and was approved by a local research and ethics committee. Information sheets and consent forms were given to the participants after the nature and consequences of the study were explained.

### Participants

For the primary aim to investigate differences between groups, ffERG responses were recorded on 68 participants with albinism, 43 with IIN, and 24 healthy controls (Table 1A). Data were available to measure OP responses in 45 participants with albinism, 35 with IIN and 15 healthy controls (Table 1B) (OPs were investigated at a later point in the study because of the emerging story of starburst amacrines involvement in studies of the FRMD7 gene (see Yonehara et al., 2016, reference 15).) For the secondary aim, eighteen participants with IN (15 with albinism and 3 with IIN) (Table 1C) were included for the within subject comparison at the null region and away from the null region. These participants were selected from a pool of individuals whose horizontal null region had been characterized in previous studies.^21^ Individuals with marked null region, defined as at least a two-fold increase in nystagmus intensity when participants held gaze away from their null region, were identified and recruited.

**Table 1.** Demographic Data of Patients With Albinism, IIN, and Healthy Controls in the Three Experiments

		Age				
Participant Group	No.	Min	Max	Mean	SD	Sex
(A) Comparisons of ERG responses between albinism, IIN, and controls						
Albinism	68	16.3	64.5	32.9	12.8	M = 41, F = 27
IIN^*^	43	16.7	65.1	36.2	12.3	M = 29, F = 14
Controls	24	18.7	77	34.8	13.4	M = 14, F = 10
(B) Comparisons of OP responses between albinism, IIN, and controls						
Albinism	45	16.3	64.5	33.2	12.0	M = 27, F = 18
IIN^*^	35	16.7	65.1	36.9	12.3	M = 25, F = 10
Controls	15	18.7	77.0	35.7	15.8	M = 10, F = 5
(C) Comparison of ERG responses at and away from null region in participants with IIN						
Albinism (*n* = 15) and IIN (*n* = 3)	18	17.7	58.8	37.6	13.6	M = 11, F = 7

AFN, away from the null region; F, female; M, male; Max, maximum; Min, minimum.

Sample sizes, ages and numbers of males and females are shown for each comparison, that is, (A) A and B wave amplitudes and peak times; (B) OPs; (C) comparison of ERG responses at and away from null region.

* The IIN group included 12 patients with FRMD7 mutation (3 females and 9 males).

The diagnostic criteria set out by Kruijt et al.^22^ 2018 were used for albinism with confirmation (see details in Supplementary Table S1). The IIN cohort had nystagmus with no VEP crossing abnormality, no iris transillumination, and only grade 1 or no fovea hypoplasia on OCT. Healthy controls had normal visual function (visual acuity of >0.1 logMAR, color vision, stereopsis) and low refractive errors (within +3.0 and −3.0 diopters [D]). Participants with any known ophthalmological or neurological diseases were excluded.

### ERG

A Ganzfeld flash ERG (Nicolet Spirit, Quebec, Canada) and DTL electrodes, which contacted the corneal margin, were used. Participants were dilated with cyclopentolate 0.5% and underwent 20 minutes of dark adaptation and 10 minutes light adaptation before the flashes were applied. The strength of the stimulus increased from a dim white flash of 0.006 cd·s·m^−2^ by 0.25 log units 12 increments, up to 1.9 cd·s·m^−2^ (the standard flash [SF]) to detect rod responses and combined rod-cone responses under scotopic condition. The SF stimulus was used to obtain the pure cone responses under photopic conditions.

For the primary aim, we followed our standard clinical procedure in that poor quality waveforms with an obvious artefact were rejected and re-recorded to obtain measurable waveforms up to a maximum of five attempts. For the secondary aim, because we were interested in the effect of nystagmus on the success rate of recording, five attempts were always made for each eye regardless of the quality of the recordings. Measurable ERG waveforms were characterized by identifiable amplitudes and consistent latencies ensuring reliability. Stability, a high signal-to-noise ratio, and appropriate responses to stimuli were also considerations for acceptable recordings. The a-and b-wave amplitudes and peak times were measured semiautomatically using the built-in software in the Nicolet Spirit device to minimize the inaccuracy (Supplementary Fig. S1). Filter setting were set according to International Society for Clinical Electrophysiology of Vision–recommended standards (100–300 Hz when recording OPs). The peak-to-trough measurements were used to obtain amplitudes of OPs.^23^

### Eye Movement Recordings

For the secondary aim, binocular eye movements were recorded using an EyeLink II pupil tracker (500 Hz, SR Research, Osgoode, Canada) while viewing an LCD monitor (Acer AL2202W, Taipei, Taiwan). Fixation targets were followed along the horizontal meridian at intervals of 3° from −30° to +30° moving every 5 seconds to determine the null region. Data were imported into Spike 2 software (Version 6.15, Cambridge Electronic Design Ltd, Cambridge, UK) for offline calibration and analysis. Data at each fixation point were analyzed after excluding blinks to derive nystagmus amplitude, frequency, and intensity.

### Effect of Nystagmus of ffERG Responses

For the secondary aim, ffERG responses were obtained at the null region and away from null region (i.e., where the gaze angle of the nystagmus was at least twice as intense). An InertiaCube BT (Thales Visionix Inc., Billerica, MA, USA) head posture device was used to set the null region and away from the null region positions by moving the head and keeping the eyes fixed on the Ganzfeld ERG fixation target (for example see Supplementary Figs. S2A, S2B).

### Statistical Analysis

Data analyses were done using R (http://www.R-project.org; accessed 18 Aug 2018).^24^ Responses for the left and right eyes were averaged. Two-way ANOVAs were used to investigate differences in ffERG responses between the three cohorts including age as a factor. Amplitude data for a-wave, b-wave measurements, and OPs were log transformed prior to analysis since data more closely approximated to normal distributions.

For the secondary aim, the feasibility of ffERG responses at the null region and away from the null region was assessed based on success and failure rates of five attempts with each eye, with success defined as good quality waveforms with measurable a- and b-wave amplitudes and peak times. For example, two of five successful recordings for the left eye and three of five for the right eye would be a 50% success rate for a participant. Success rates at null region and away from the null region were compared using paired *t* tests for parametric data and Wilcoxon tests for nonparametric data.

## Results

### Original Recordings

Original recordings are shown in Supplementary Figure S3A for representative participants with albinism, IIN, and a control. Supplementary Figure S3B provides recordings of some examples of artefactual data such as would be deemed failures for the secondary aim of the study.

### Primary Aim: Comparison of ERGs in Participants With Albinism, IIN, and Healthy Controls

Outcomes of ANOVAs for a- and b-wave amplitudes and peak times are shown in Table 2 including group (albinism, IIN or control) age and Group × Age as factors. Medians and quartiles for a- and b wave amplitudes and peak times in each group are also provided in Supplementary Table S2A.

**Table 2.** Outcomes of ANOVAs for a- and b-wave Amplitudes and Peak Times Including Group (Albinism, IIN, or Control), Age, and Group × Age

	Group		Age		Group × Age	
	F	*P* Value	F	*P* Value	F	*P* Value
Photopic						
a-Wave amplitude	**9.723**	**<0.0001**	3.925	0.05	**11.525**	**<0.0001**
b-Wave amplitude	**4.886**	**0.009**	1.98	0.162	**3.638**	**0.029**
a-Wave peak time	0.825	0.441	**4.422**	**0.037**	1.736	0.180
b-Wave peak time	0.373	0.689	**7.776**	**0.006**	1.617	0.203
Scotopic DIM						
b-Wave amplitude	1.874	0.159	0.177	0.675	1.658	0.196
a-Wave peak time	1.746	0.180	3.245	0.075	1.746	0.180
b-Wave peak time	0.11	0.896	**4.517**	**0.036**	0.201	0.818
Scotopic SF						
a-Wave amplitude	0.855	0.428	**6.963**	**0.009**	1.876	0.157
b-Wave amplitude	1.189	0.308	0.06	0.807	0.999	0.371
a-Wave peak time	1.975	0.143	**12.628**	**<0.0001**	1.595	0.207
b-wave peak time	1.511	0.225	2.484	0.118	0.885	0.415

Boldface entries indicate statistical significance.

#### Photopic Conditions With SF

**Figure 1.**
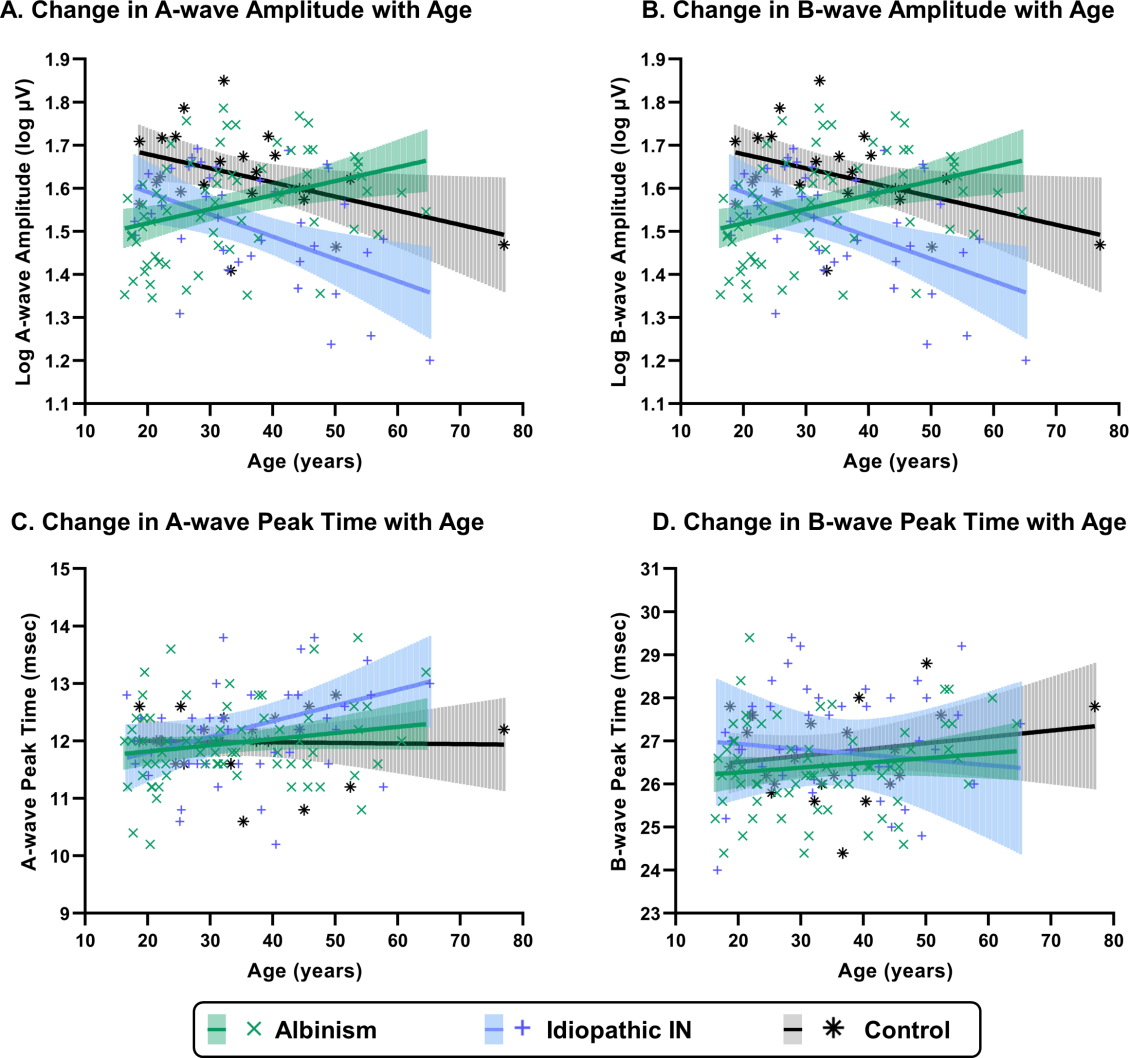
Plots of ERG amplitudes and peak times with age under photopic conditions with SF. The results indicate that albinism and IIN groups have significantly smaller a- and b-wave amplitudes compared with healthy controls under photopic conditions. The albinism group presented shorter b-wave peak times compared with the IIN group. However, the two patient groups showed no significant differences in photopic a- and b-wave peak times when compared with controls.

Plots of photopic a and b wave amplitudes and peak time against age are shown in Figure 1. A strong significant interaction between group and age for photopic a-wave amplitude, *F* = 11.53, *P* < 0.0001, was caused by ffERG responses increasing with age in albinism but decreasing with age in IIN and controls (Fig. 1). A similar pattern led to a weak interaction between group and age for b-wave amplitude, *F* = 3.64, *P* = 0.029.

Age-adjusted photopic a- and b-wave amplitude were consistently lower in the IIN group compared with controls, *F* = 14.23, *P* < 0.0001 and *F* = 17.10, *P* < 0.0001, respectively. Areas under curves for receiver operator curves comparing photopic a- and b-wave amplitudes, adjusted for age, in the IIN group compared with controls were above 0.75 (a-wave: 0.78; 95% confidence interval, 0.66–0.90; b-wave: 0.77; 95% confidence interval, 0.65–0.89).

**Figure 2.**
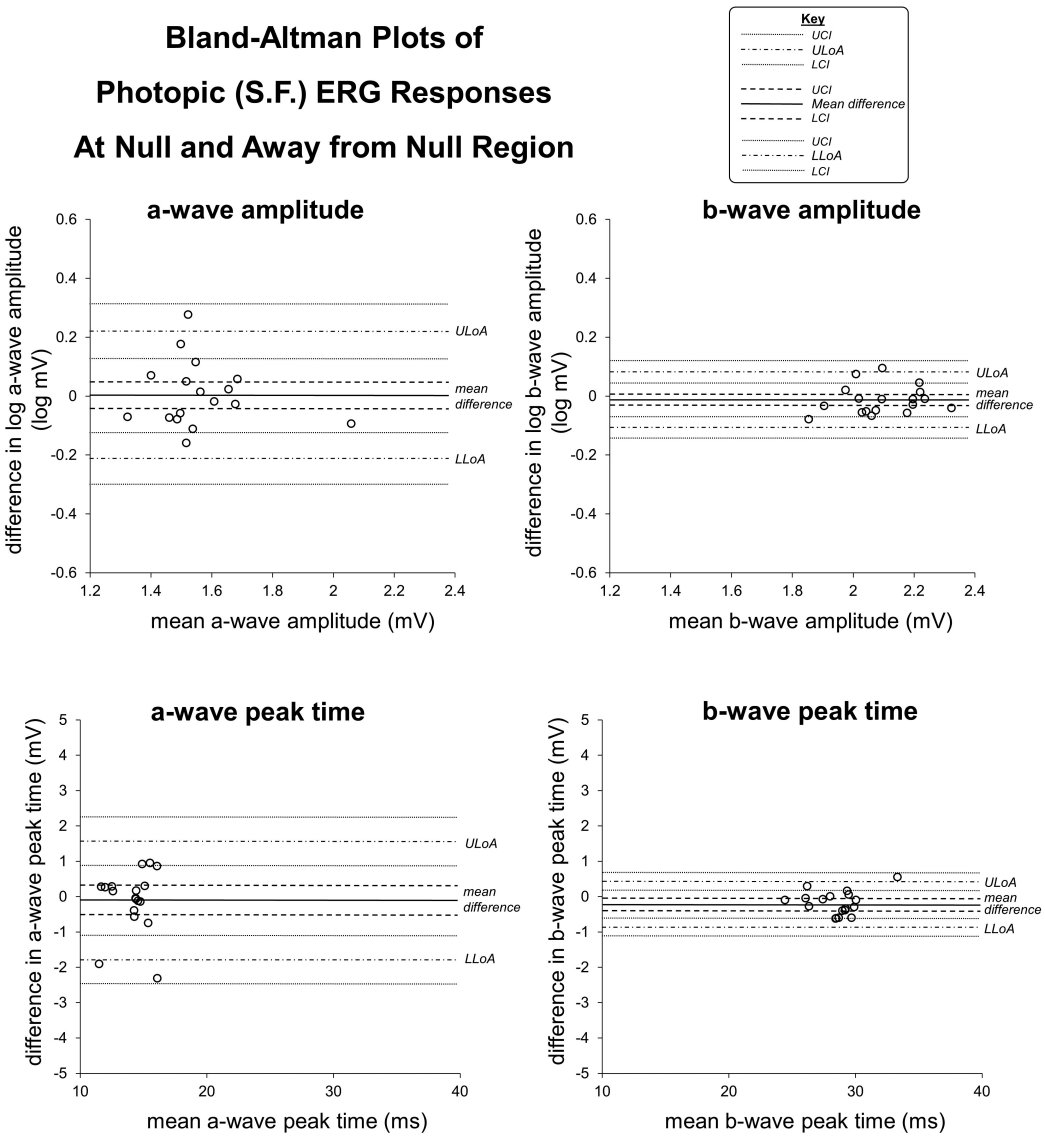
Bland–Altman plots showing the difference in within subject ERG responses tested at the null region and at the position away from the null region under photopic condition. *Solid lines* indicate the mean difference with *dashed lines* showing the 95% confidence intervals. The *dashdotted* lines are the upper and lower limits of agreement with the dotted lines showing the 95% confidence intervals. LLoA, lower limits of agreement; UloA, upper limits of agreement.

In contrast, photopic a- and b-wave amplitude in albinism were smaller than controls at an early age and larger than controls later in life. Photopic a-wave amplitudes demonstrated a significant increase with age (*r* = 0.35, *P* = 0.035, slope = 0.00325 log mV increase per year) in the albinism group but changes in photopic b-wave amplitudes with age were not significant (*P* = 0.148). An exploratory subanalysis comparing *FRMD7*-associated IIN (*n* = 12) with controls (*n* = 24) is included in Supplementary Table S3. Age was a significant factor for photopic b-wave peak times, *F* = 7.77, *P* = 0.006, with a borderline effect seen for a-wave peak times (Table 2, Fig. 2).

#### Scotopic Conditions

There was no difference between albinism, IIN and controls groups under scotopic conditions with dim and SFs for a- and b-wave amplitude and peak time measures (Table 2). However, age was a significant factor most notably for a-wave amplitudes and peak times under scotopic SF, *F* = 6.96, *P* = 0.009 and *F* = 12.63, *P* < 0.001, respectively, with amplitudes decreasing with age and peak times increasing with age.

#### Ratio of Cone/Rod Responses

The ratio of rod responses (scotopic [dim] b-wave amplitude) over cone responses (photopic [SF] b-wave amplitude) was elevated in the control group compared with both the albinism and IIN groups, ANOVA: group effect: *F* (2, 94) = 1.75, *P* = 0.017; post hoc: albinism compared with controls: *P* = 0.015; IIN compared with controls: *P* = 0.015.

#### Comparisons of OPs Peaks Measurements Between Albinism, IIN, and Healthy Controls

There were no statistically significant differences between the three groups for OP amplitude or peak time measures, combined models: *F* = 1.77, *P* = 0.176; *F* = 0.58, *P* = 0.56, respectively, although age was a significant factor, *F* = 7.57, *P* = 0.007; *F* = 7.51, *P* = 0.007, respectively. The only exception was O1 amplitudes where age-adjusted O1 amplitudes were consistently higher in the albinism group compared with the other two groups, *F* = 6.63, *P* = 0.002 (Supplementary Fig. S4). Medians and quartiles for O1, O2, O3 and O4 measures are provided in Supplementary Table S2B.

### Secondary Aim: Effect of Nystagmus on ffERG Responses

The percentage of measurable waveforms were assessed for 5 repeats of ffERG responses at the null region (mean 3°) and away from the null region (mean 18°) (Supplementary Fig. S4 and Table S4) for each eye. Under the scotopic condition, with dim flash the success rate was 50% at null region dropping to 20% away from the null region, *t*(17) = 4.86, *P* = 0.0001; and with SF 90% at null region compared with 50% away from the null region (Wilcoxon: *P* = 0.0030). Under light-adaptation conditions with SF, the success rate was 85% at the null region and 50% away from the null region, *t*(17) = 2.99, *P* = 0.0082.

Photopic b-wave peak times were slightly but significantly slower when recorded at the null region compared with away from the null region (mean difference = 0.67%, *P* = 0.027) (Table 3; Bland–Altman plots are shown for the photopic condition in Fig. 2). Under scotopic conditions with dim flash, a- and b-wave mean amplitudes and peak times were similar at the null region and away from the null region but standard deviation where higher away from the null region compared with at the null region. Under scotopic conditions with SF, a-and b-wave amplitudes were significantly larger at the null region compared with away from the null region (a-wave amplitudes: 17.05% larger, *P* = 0.023; b-wave amplitudes: 10.07% larger, *P* = 0.018).

**Table 3.** Paired Comparison of ERG Responses Tested at the Null Region and AFN

Parameters	Null Point	AFN	Difference	Upper LoA	Lower LoA	Percentage Difference	*P* Value
Photopic SF							
a-Wave amplitude (log µV)	1.56 ± 0.16	1.56 ± 0.15	0.000	0.196	−0.196	−0.32%	0.856
b-Wave amplitude (log µV)	2.10 ± 0.11	2.08 ± 0.12	0.010	0.088	−0.068	0.69%	0.221
a-Wave peak time (msec)	14.1 ± 1.45	14.0 ± 1.50	0.120	1.747	−1.507	0.85%	0.559
b-Wave peak time (msec)	28.6 ± 1.89	28.4 ± 1.93	0.190	0.817	−0.437	0.67%	0.027
Scotopic DIM							
b-Wave amplitude (log µV)	2.39 ± 0.14	2.40 ± 0.20	−0.010	0.284	−0.304	−0.43%	0.817
a-Wave peak time (msec)	39.1 ± 5.70	39.9 ± 4.37	−0.760	9.295	−10.815	−1.95%	0.615
b-Wave peak time (msec)	94.5 ± 15.4	99.9 ± 19.2	−5.350	15.426	−26.126	−5.67%	0.105
Scotopic SF							
a-Wave amplitude (log µV)	2.26 ± 0.12	2.17 ± 0.13	0.080	0.315	−0.155	3.63%	0.023
b-Wave amplitude (log µV)	2.57 ± 0.10	2.52 ± 0.11	0.040	0.177	−0.097	1.86%	0.027
a-Wave peak time (msec)	19.1 ± 3.01	19.9 ± 2.96	−0.720	4.062	−5.502	−3.79%	0.268
b-Wave peak time (msec)	48.9 ± 4.03	50.1 ± 5.01	−1.140	5.132	−7.412	−2.34%	0.173

AFN, away from the null region.

The data show are mean ± SD. The data in the percentage difference column are calculated using the formula % difference = (Data (Null) – Data (AFN))/(Data (Null)) × 100%. The positive difference means that the response obtained from the null region is larger (amplitude) or longer (peak time) than the data collected from the region AFN. The negative data means the data gained at the null region is smaller (amplitude) or shorter (peak time) compared with the data from the position AFN. *P* values are not adjust across statistical models.

LoA, limits of agreement.

## Discussion

In this study, a substantially larger sample size of participants with albinism (*n* = 68) and IIN (*n* = 43) have been compared with controls (*n* = 24) than in previous studies (see Supplementary Table S5 for a comparison). Adult participants were recorded using DTL electrodes which contacted the corneal margin and have a better signal-to-noise ratio than skin electrodes, which were mainly used in the previous studies (Supplementary Table S5).^8^^–^^10^ Using this robust methodology, we detected previously unreported differences in age-adjusted ffERG responses in IIN compared with healthy controls (*P* < 0.0001), with responses in both groups decreasing with age. In contrast, photopic a-wave amplitudes increased with age in albinism (*P* = 0.0035) with the result that difference compared with the control group depended on age. B-wave amplitudes did not change significantly.

Nystagmus eye movements were shown to consistently influence the success rate of recordings which was 30% to 40% better at the null region compared with away from the null region. However, increased nystagmus intensity is unlikely to have a clinically significant effect on photopic or SF scotopic amplitudes and peak times, given the small percentage differences in ERG responses at the null and away from the null region, as shown in Table 3.

### ffERG Responses in Albinism

The interaction terms of group and age was highly significant for photopic a- and b-wave amplitude owing to amplitudes increasing with age in the albinism group in contrast with the IIN and control groups, which both decreased with age (Fig. 1). These finding highlights the importance of accounting for age in detecting abnormalities in INS groups. In agreement with previous literature, age influenced numerous ffERG responses with the strongest effects observed in b-wave and OP peak times, which become increasingly delayed with age.^25^ Interestingly, adjusting for age identified elevated O1 amplitudes of OPs.

It is not clear why there are different patterns in age-related changes in photopic amplitudes in albinism compared with the other two groups. Recently, Lee et al.^26^ used hand-held OCT to document changes in retinal architecture in the early years of life, demonstrating that slow changes continue to occur up to 12 years of age.

Overall, differences in the albinism cohort compared with controls were mild in comparison with the IIN group for participants <40 years of age (Fig. 1) despite structural retinal changes being more significant. A recent study by Kuht et al*.*,^27^ for example, reports a spectrum of foveal hypoplasia associated with OCA mutations whereas OA mutations always lead to severe with grade 3 or 4 hypoplasia. In contrast, *FRMD7* mutations are associated with very mild foveal hypoplasia of grade 1 (16%) or none at all (84%).^27^ The appearance of the optic nerve with enlarged rims and circumpapillary retinal nerve fiber layer thinning is also more severe in albinism.^7^ Several histological studies also highlight abnormalities in photoreceptor populations including 30% reduction in rods in mammals,^2^^,^^28^ and reduced numbers of cones, especially long-wave sensitive cones in albino mice.^29^

Given the striking retinal changes in albinism, it is surprising ffERG responses are not affected more adversely. Hypopigmentation may explain the discrepancy with iris transillumination, permitting more light into the eyes to stimulate the retina and with fundus hypopigmentation also increasing light scatter. In previous studies, hypernormal ERG responses in animals^30^^,^^31^ and participants with albinism have been reported^11^^–^^13^; however, we did not observe this pattern.

### ffERG Responses in IIN

Reduced photopic a- and b-wave amplitudes in IIN, either recorded directly and adjusted for age or expressed relative to scotopic response, indicate abnormal physiological response in the retina that parallel the structural abnormalities previously characterized using OCT.^14^^,^^32^ IIN participants with *FRMD7* mutations have significantly shallower foveal pits, smaller optic discs and cups, and have decreased retinal nerve fiber layer thickness compared with controls. Our findings are unlikely to be of diagnostic use because, including a 5% margin of error in a clinical setting, all the IIN group in this study had normal b-wave amplitude and peak times, the most important diagnostic indicators.

Abnormal ffERG amplitudes in the IIN group have not been reported previously and contrast with the normal responses described in previous studies.^8^^–^^10^ This finding may relate to the more accurate and reliable methodology used in this study. Improvements in methodology include (i) a larger sample size, (ii) use of DTL electrodes with higher signal-to-noise ratio compared with skin electrodes, (iii) testing with pupils dilated, and (iv) testing in adults with better cooperation than children.

The IIN group did not show changes in amplitudes under scotopic conditions and the ratios of ffERG responses of cones to rods were significantly lower in the IIN group compared with the control cohort. This finding may indicate more severe deficits in the cone system compared with the rod system in IIN, either because of connectivity or because cone numbers are reduced. Thomas et al.^14^ observed significant thinning of the outer segment layer in central macular in IIN participants with *FRMD7* mutations compared with controls.

Amacrine, bipolar, and/or ganglion cells are thought to be involved in the generation of OPs,^16^^–^^18^ and recent studies in mice have reported abnormal physiological responses in starburst amacrine cells.^15^ However, we observe no significant differences in OP amplitudes or peak times in IIN compared with controls.

Recently, *FRMD7* mutations have been show to influence horizontal motion selectivity in mouse models, which is thought to be mediated by the inhibitory effect of starburst amacrine cells upon retinal ganglion cells.^15^ A possible mechanism is that *FRMD7* mutations disrupt spontaneous retinal waves occurring during visual development,^33^ which mimic optic flow occurring later in life.^34^ Interestingly, in addition to the changes observed in the wider IIN group compared with controls, for 12 participants shown to have *FRMD7*-associated IIN, changes in timing of retinal responses are also seen, including slower photopic a- and b-waves and quicker scotopic b-waves with SF. These may be indicative of timing-related changes in wiring of retinal circuitry but would need confirmation in a larger sample size.

### Effect of Nystagmus Eye Movements of ffERG Responses

Repeated measures comparisons were made at the null region and away from the null region using participants with clear null regions to generate different degrees of nystagmus. Although statistically significant differences were observed for photopic b-wave peak times, the mean difference was <1% and, therefore, unlikely to be clinically significant. Under scotopic condition with SF, a-and b-wave amplitudes were significantly larger at the null region compared with away from the null region (17.05% and 10.07%, respectively).

More important, nystagmus eye movements influenced the success of gaining measurable responses, which were 35%, 40%, and 30% better under photopic conditions and scotopic conditions with SF and dim flash, respectively. This finding was especially important for scotopic conditions with dim flash, where success decreased to 20% in the away from the null region position. From a practical point of view, determining the approximate null region before recording ffERG responses and using head position to measure ffERG responses at the null region could be implemented as a useful strategy for gaining consistent responses.

### Limitations

For the primary aim, we recorded ffERG response rather than focal ERGs, which, given the OCT foveal changes described,^35^ would be important to test in future studies. For the secondary aim, only 3 of the 18 participants included had IIN with the remaining being participants with albinism. This factor could introduce bias, and it would be useful to confirm this question on a larger sample of individuals with IIN. IN cohorts were mostly defined based on phenotype owing to limited genetic data. With the recent availability of nystagmus panels, future studies can explore ERG responses in specific genetic subtypes. Because ERG studies generate many parameters, this necessitates a large number of statistical comparisons. We have only corrected for multiple comparisons within individual statistical models so the *P* values need to be interpreted accordingly with some caution.

## Conclusions

We find significant changes in ffERG photopic responses in both albinism and IIN groups that are unlikely to be of diagnostic use but raise caution over whether ffERG responses in these groups can be considered as normal. They add to a growing body of evidence confirming that, not only the albinism group, but also the IIN group, have global structural and functional retinal deficits. The effect of nystagmus eye movements is more important in influencing the success of achieving responses rather than on the impact of the measures directly since all recorded values fall in normal ranges.

## Supplementary Material

Supplement 1

Supplement 2
