# Supplementary material for: ERG Responses in Albinism, Idiopathic Infantile Nystagmus, and Controls
Source: Invest Ophthalmol Vis Sci. 2024 Apr 4;65(4):11. doi: 10.1167/iovs.65.4.11 (PMC10996992; doi:10.1167/iovs.65.4.11)
Supplement: Supplement 1 [file iovs-65-4-11_s001.docx]

**Supplementary Material**

**Supplementary Figure S1: Amplitude and peak time measurements derived from original electroretinography (ERG) waveforms under photopic and scotopic conditions for participants with albinism, idiopathic infantile nystagmus (IN) and controls.**

**Supplementary Figure S2: Method used to determine the null region in participants with infantile nystagmus (IN) (albinism and idiopathic IN) to investigate the effect of nystagmus intensity on electroretinography (ERG) responses.**

**Supplementary Figure S3: Original recording of electroretinographical responses for primary and secondary aims**

**Supplementary Figure S4: Change in O1 amplitude with age for each group**

**Supplementary Figure S5: Comparison of electroretinography (ERG) testing success rates between tested at null region and away from null (AFN) in patients with infantile nystagmus**

**Supplementary Table S1: The diagnostic criteria for albinism**

**Supplementary Table S2: Medians and quartiles for: (A) a- and b wave amplitudes and peak times and (B) oscillatory potential amplitudes and peak times.**

**Supplementary Table S3: Outcomes of linear mixed models for: a- and b- wave amplitudes and peak times including groups (idiopathic infantile nystagmus with *FRMD7* mutation or control).**

**Supplementary Table S4: Comparison of electroretinography (ERG) testing success rates between tested at null region and away from null (AFN) in patients with infantile nystagmus**

**Supplementary Table S5: Comparisons of electroretinography (ERG) findings between the current study and previous studies**

**Supplementary Figure S1: Amplitude and peak time measurements derived from original electroretinography (ERG) waveforms under photopic and scotopic conditions for participants with albinism, idiopathic infantile nystagmus (IN) and controls.**

A- and B-wave amplitudes and peak times were measured where possible for each waveform, where:

- A-wave peak times (La) were measured from the stimulation onset to the trough of the a-wave
- B-wave peak times (Lb) were measured from the stimulation onset to the peak of the b-wave.
- A-wave amplitudes (Aa) were measured from a preceding peak to the trough of the a-wave.
- B-wave amplitudes (Ab) were measured from the trough of a-wave to the peak of b-wave.

The waveforms and measurements of participants with albinism, idiopathic IN and controls are presented in upper, middle and lower rows, respectively.

Traces from participants with albinism and idiopathic IN demonstrate slightly more noise compared to the control group.


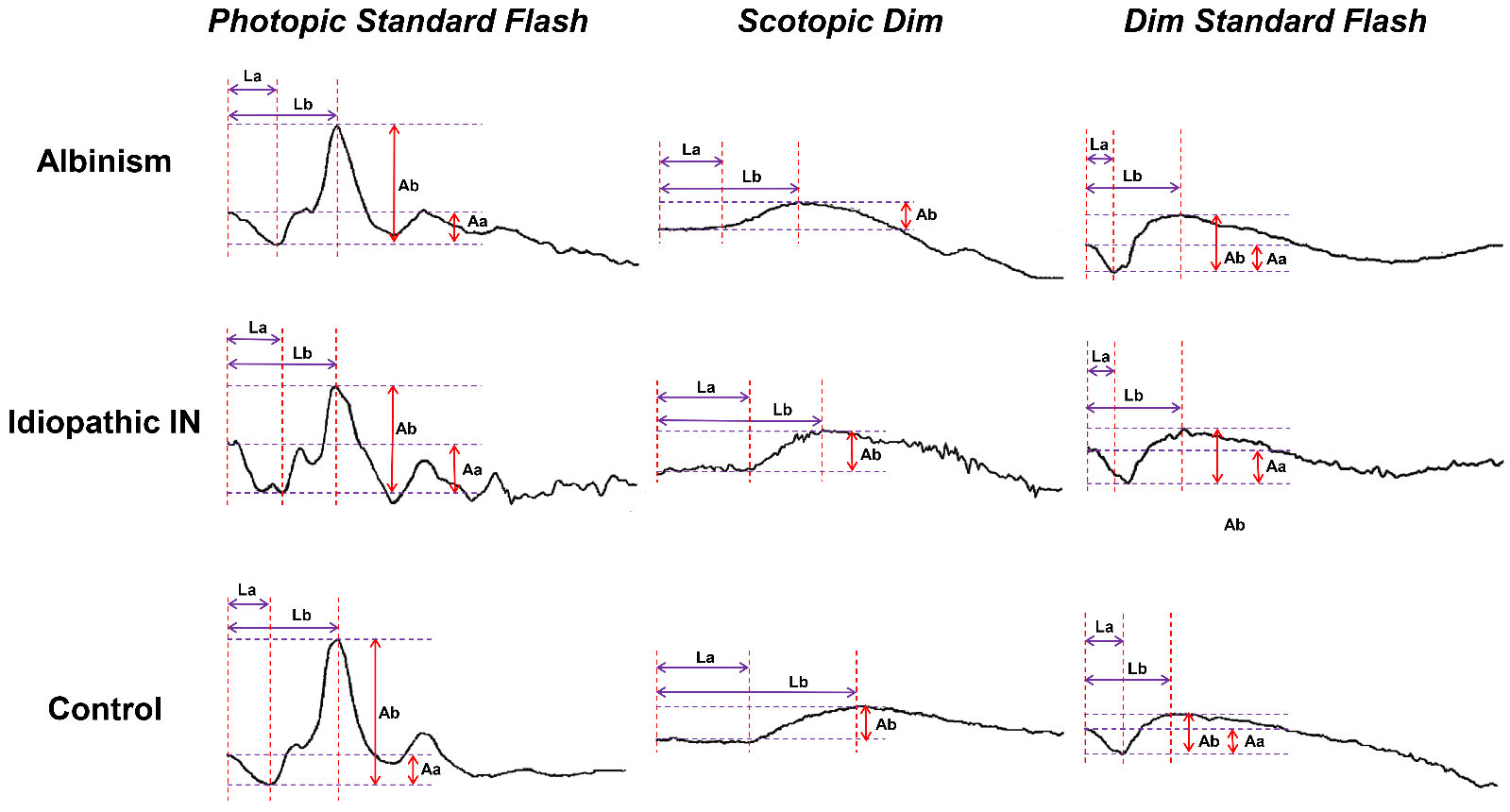


**Supplementary Figure S2: Method used to determine the null region in participants with infantile nystagmus (IN) (albinism and idiopathic IN) to investigate the effect of nystagmus intensity on electroretinography (ERG) responses.**

In (A) an original recording of horizontal eye movements is shown for a participant with idiopathic IN. The participant was requested to fixate on a stationary target that moved every 7 seconds from 30°on the left (downwards on the trace) to 30° on the right (upwards on the trace) in 3° steps.

In (B) analysis of the intensity of the nystagmus (amplitude x frequency) at each gaze angle is used to derive the region of lowest nystagmus intensity (null region) which is compared to a region where the nystagmus is more intense (away from null region).


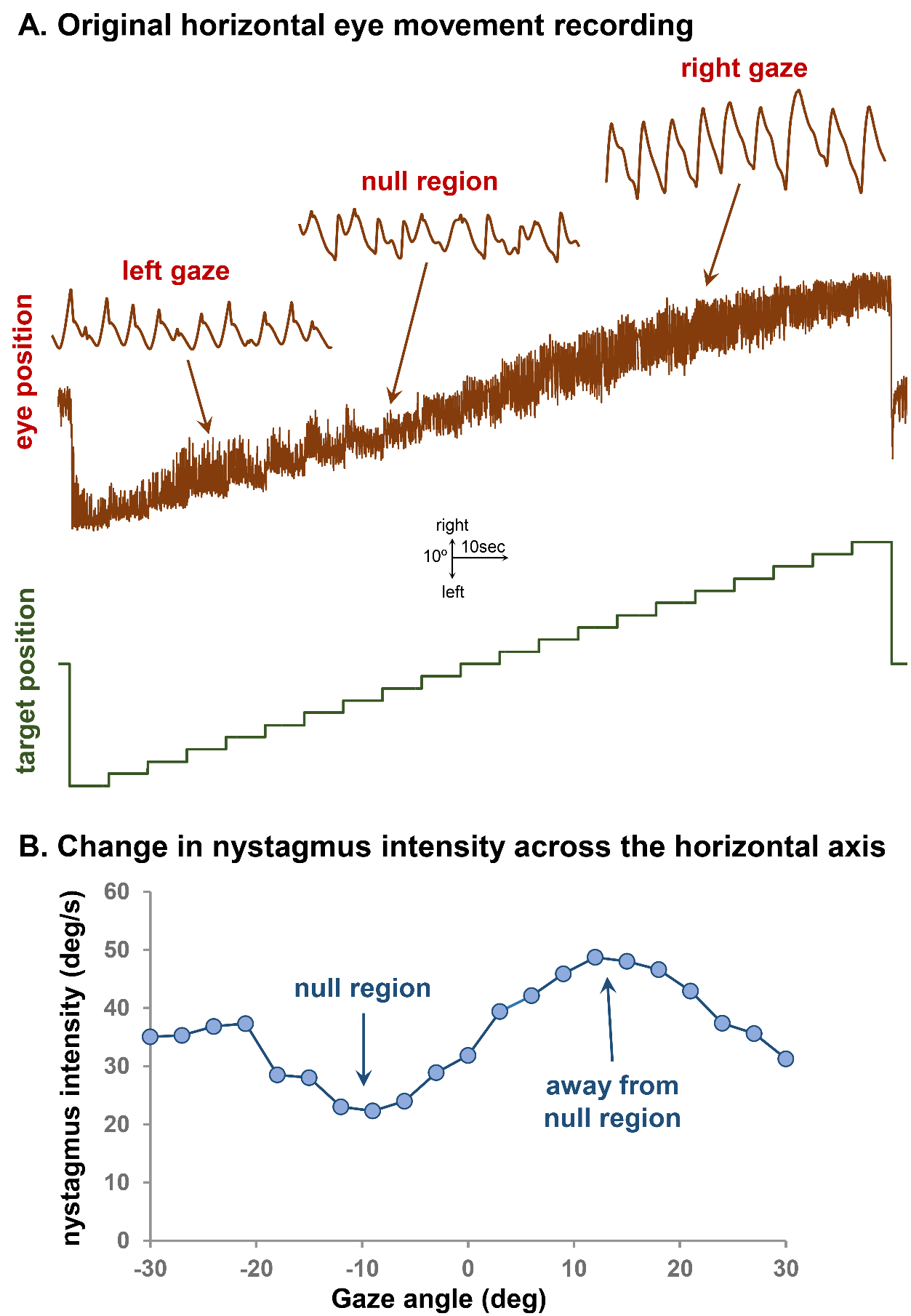


**Supplementary Figure S3: Original recording of electroretinographical responses for primary and secondary aims**

**A.** Original recordings of electroretinographical responses from representative participants with idiopathic infantile nystagmus (IN), albinism and a healthy control as stimulus intensity increases. Two sets of example data are shown below for each group (albinism, IIN and controls). The waveforms in set A (left) are of high quality and measurable. In contrast, some of the waveforms in set B (right) show baseline drifts which are not measurable and hence the data was excluded (marked using red crosses).

**
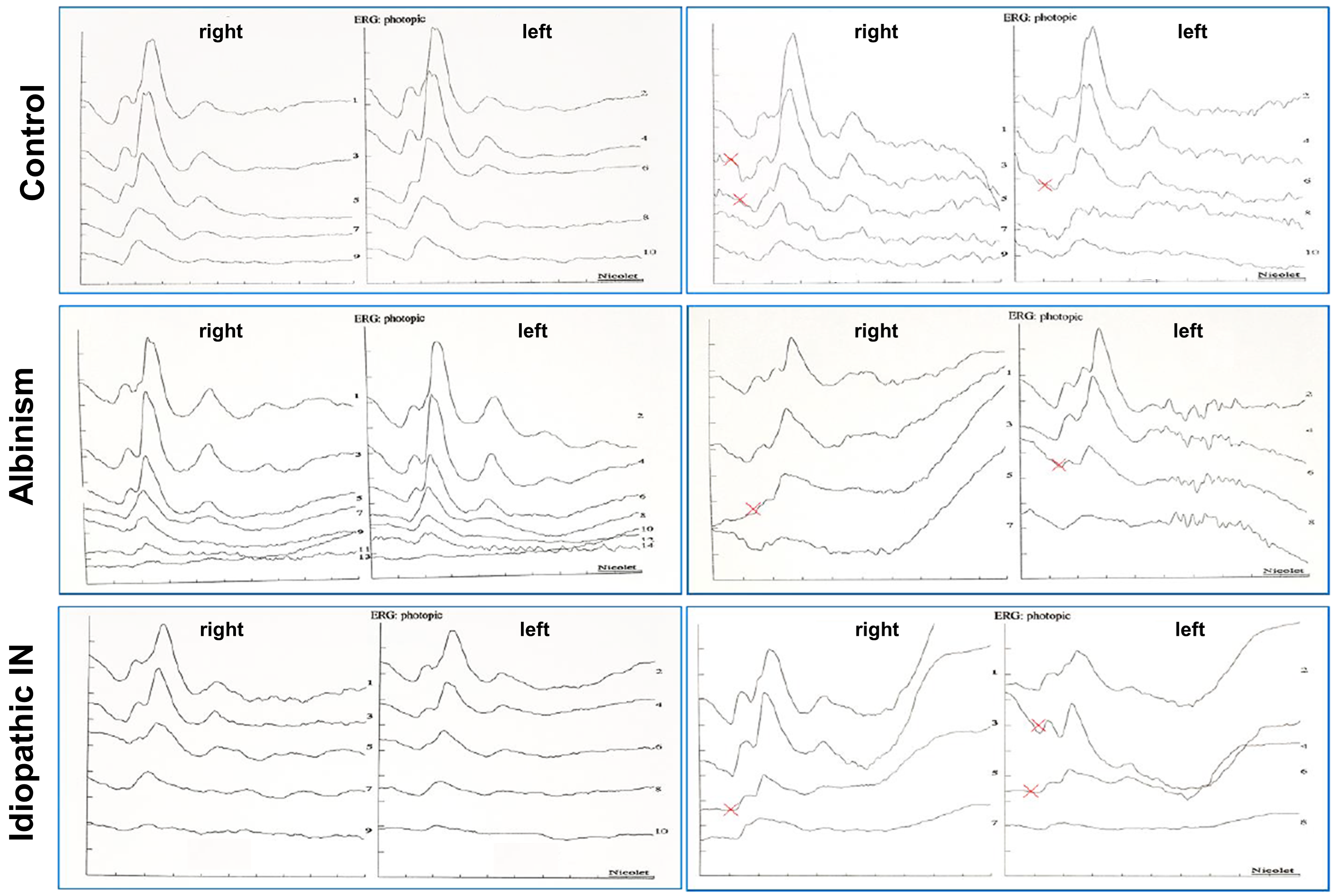
**

**B.** Original recordings of electroretinographical responses for the secondary aim. The waveforms were collected from a patient with idiopathic infantile nystagmus under scotopic conditions with 12 strengths of stimulus flashes. Artefacts caused by nystagmus affect the ERG measurements. The waveforms with a blue cross could not be measured. The a- and b-waves in measurable waveforms were marked using red vertical lines. The second step of light strength is dim stimulus flash and the twelfth step is the S.F. (1.9 cd∙s∙m-2) in this study.


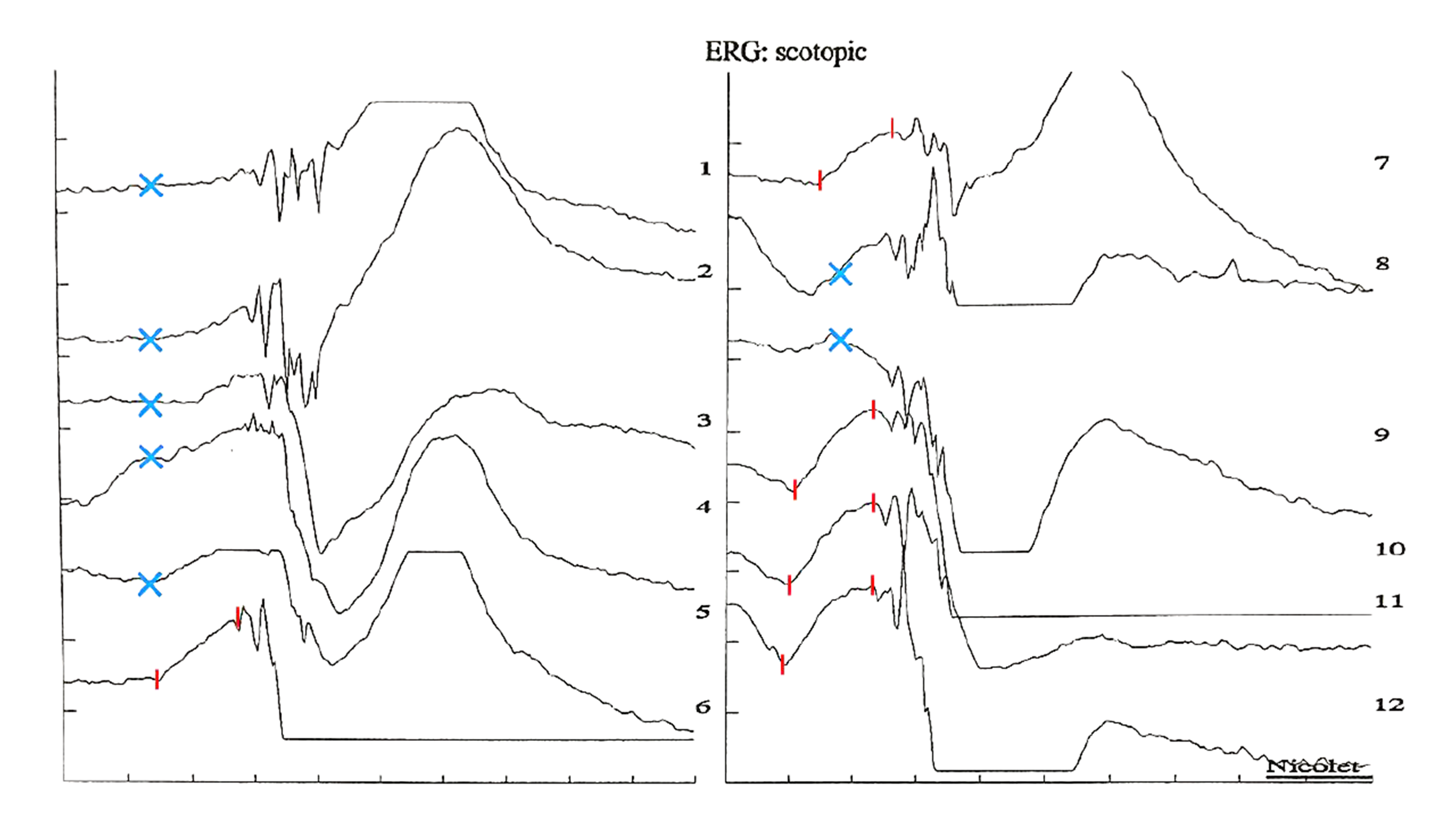


**Supplementary Figure S4: Change in O1 amplitude with age for each group**

The amplitudes of oscillatory potentials O1 peak and age (years) were plotted to find O1 amplitude changes with age in the three cohorts (IIN in blue, albinism in green and controls in red). The graph illustrates that the line representing the albinism group was parallel to the best-fit line produced by the data collected from controls but shifted up. The best-fit line in the IIN group completely overlapped with the best-fit line in the control group. The shifted-up best-fit line in the albinism cohort indicates increased O1 amplitudes compared to the IIN and control groups in the same ages.


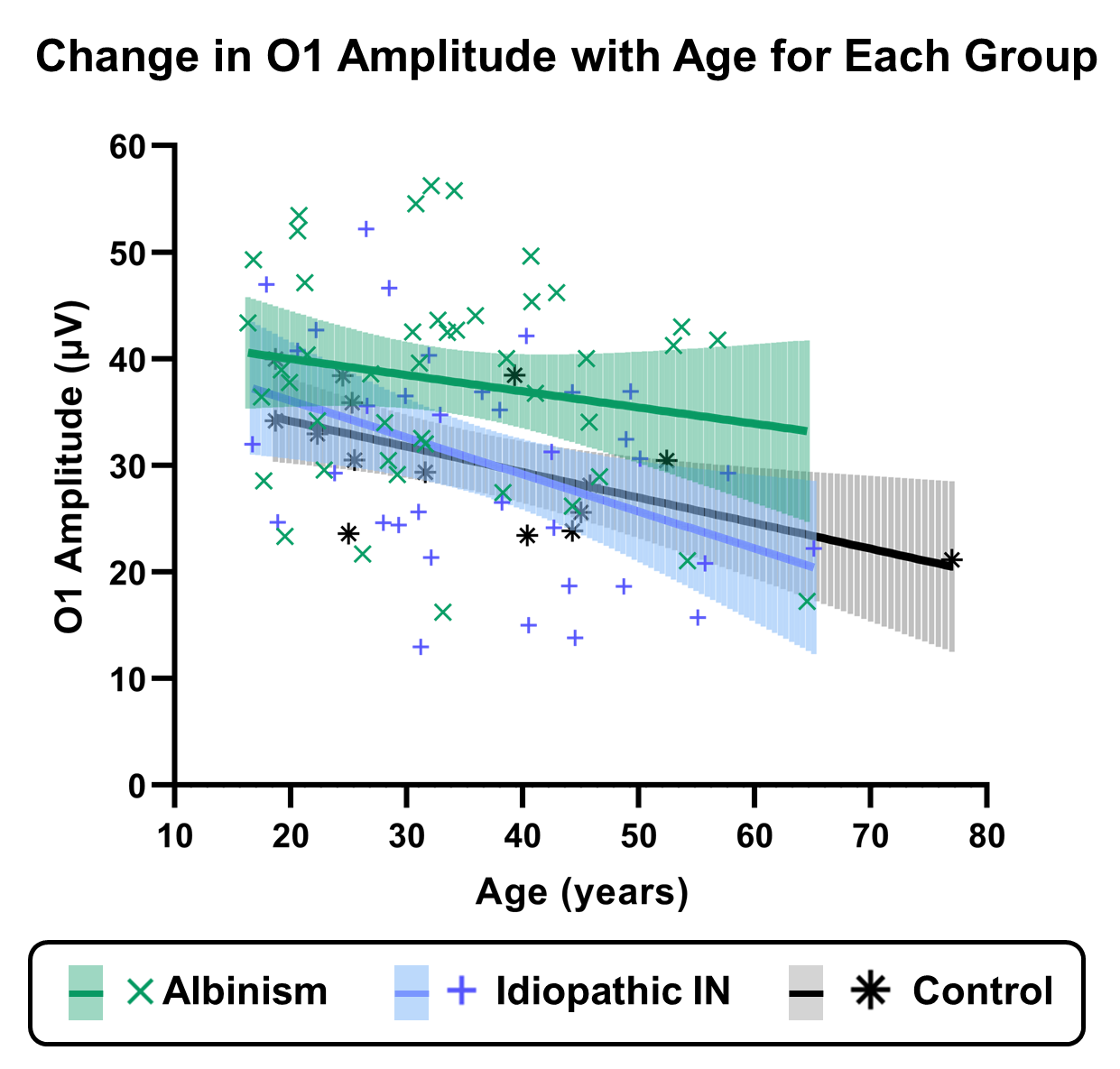


**Supplementary Figure S5: Comparison of electroretinography (ERG) testing success rates between tested at null region and away from null (AFN) in patients with infantile nystagmus**

S.F.: standard flash; AFN: away from null.

##
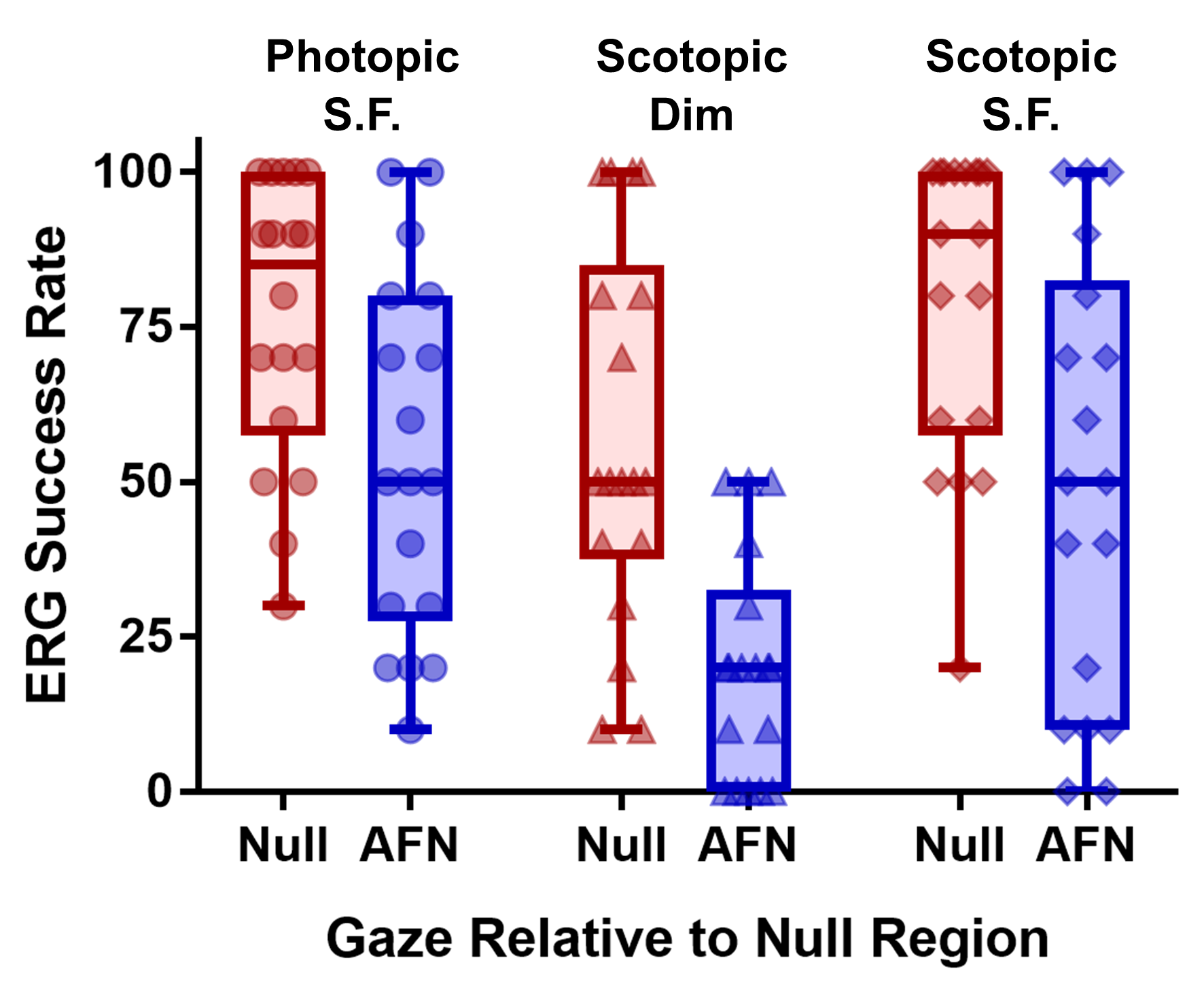


**Supplementary Table S1 The diagnostic criteria for albinism**

The diagnostic criteria was used for albinism with confirmation of either three major criteria or two major and two minor criteria for a positive diagnosis.^1^ VEP: visual evoked potentials

| **The diagnostic criteria for albinism** | |
| --- | --- |
| **Major criteria** | **Minor criteria** |
| Grade 2 or more foveal hypoplasia | Nystagmus |
| Misrouting confirmed using visual evoked potentials (VEP) | Hypopigmentation of skin and hair |
|  | Grade 1 fundus hypopigmentation |
| ocular hypopigmentation (either iris translucency or fundus hypopigmentation grade 2 or more |  |
|  | Foveal hypoplasia grade 1 |

**Supplementary Table S2: Medians and quartiles for: (A) a- and b-wave Amplitudes and Peak times and (B) Oscillatory Potential Amplitudes and Peak times including data from right and left eyes.**

| A. a- and b-wave amplitudes and peak times | | | | | | | | | |
| --- | --- | --- | --- | --- | --- | --- | --- | --- | --- |
|  |  | **median value (lower quartile, upper quartile)** | | | | | | | |
|  |  | **Albinism** | |  | **Idiopathic IN** | |  | **Controls** | |
| **PHOTOPIC** | a-wave amplitude | 37.6 | (30.8, 44.1) |  | 33.7 | (27.1, 41.9) |  | 41.9 | (38.9, 49.3) |
|  | b-wave amplitude | 130.0 | (105.8, 157.6) |  | 118.5 | (99.9, 146.5) |  | 148.4 | (140.9, 184.7) |
|  | a-wave peak time | 12.0 | (11.6, 12.3) |  | 12.2 | (11.6, 12.8) |  | 12.0 | (11.8, 12.3) |
|  | b-wave peak time | 26.4 | (25.8, 27.1) |  | 27.0 | (26.3, 27.9) |  | 26.6 | (26.0, 27.6) |
| **SCOTOPIC DIM** | b-wave amplitude | 292.0 | (245.5, 347.7) |  | 257.0 | (224.9, 310.5) |  | 291.7 | (214.5, 365.4) |
|  | a-wave peak time | 42.0 | (39.2, 43.2) |  | 41.6 | (39.3, 43.1) |  | 40.0 | (38.8, 41.4) |
|  | b-wave peak time | 80.0 | (74.4, 86.4) |  | 79.8 | (74.4, 82.4) |  | 80.0 | (77.0, 87.2) |
| **SCOTOPIC STANDARD FLASH** | a-wave amplitude | 263.7 | (231.5, 306.9) |  | 260.7 | (201.2, 278.1) |  | 242.9 | (202.3, 289.8) |
|  | b-wave amplitude | 461.2 | (408.2, 547.4) |  | 431.4 | (387.3, 507.4) |  | 458.3 | (360.5, 595.5) |
|  | a-wave peak time | 19.2 | (18.4, 20.0) |  | 19.6 | (18.4, 20.0) |  | 19.4 | (18.5, 20.0) |
|  | b-wave peak time | 45.6 | (43.2, 48.8) |  | 44.0 | (42.4, 46.4) |  | 45.8 | (44.6, 47.5) |

| B. Oscillatory potential amplitudes and peak times including data from right and left eyes. | | | | | | | | | |
| --- | --- | --- | --- | --- | --- | --- | --- | --- | --- |
|  |  | **median value (lower quartile, upper quartile)** | | | | | | | |
|  |  | **Albinism** | |  | **Idiopathic IN** | |  | **Controls** | |
| **AMPLITUDE** | O1 amplitude | 38.8 | (44.5, 30.0) |  | 31.1 | (37.6, 21.9) |  | 30.0 | (36.9, 24.4) |
|  | O2 amplitude | 40.0 | (53.3, 30.9) |  | 41.0 | (51.8, 28.9) |  | 44.3 | (51.4, 39.5) |
|  | O3 amplitude | 28.6 | (39.4, 18.4) |  | 26.3 | (37.6, 16.0) |  | 29.9 | (38.9, 21.7) |
|  | O4 amplitude | 25.3 | (36.1, 18.8) |  | 24.9 | (30.4, 18.0) |  | 22.1 | (32.5, 18.2) |
| **PEAK TIME** | O1 peak time | 14.9 | (14.3, 15.4) |  | 14.5 | (13.9, 15.4) |  | 14.8 | (14.4, 15.2) |
|  | O2 peak time | 21.7 | (21.0, 22.4) |  | 21.3 | (20.6, 22.3) |  | 21.4 | (20.8, 22.0) |
|  | O3 peak time | 28.7 | (27.8, 29.5) |  | 28.1 | (27.1, 29.0) |  | 28.1 | (27.2, 28.6) |
|  | O4 peak time | 36.2 | (34.8, 37.4) |  | 35.4 | (34.1, 36.7) |  | 35.1 | (34.2, 36.2) |

**Supplementary Table S3: Outcomes of linear mixed models for: a- and b- wave amplitudes and peak times including groups (idiopathic infantile nystagmus with *FRMD7* mutation or control).**

| **A- and b-wave amplitudes and peak times** | | | | | | | |  |
| --- | --- | --- | --- | --- | --- | --- | --- | --- |
|  |  | **median value (lower quartile, upper quartile)** | | | | | | **T-Test** |
|  |  |  | **Idiopathic IN with *FRMD7* Mutation** | |  | **Controls** | |  |
| **PHOTOPIC** | a-wave amplitude |  | 34.8 | (27.3, 43.3) |  | 41.9 | (38.9, 49.3) | **0.027** |
|  | b-wave amplitude |  | 129.7 | (100.7, 152.3) |  | 147.4 | (138.5, 184.7) | 0.056 |
|  | a-wave peak time |  | 12.6 | (12.1, 12.9) |  | 12.0 | (11.8, 12.3) | **0.010** |
|  | b-wave peak time |  | 27.7 | (27.0, 28.3) |  | 26.4 | (26.0, 27.5) | **0.004** |
| **SCOTOPIC DIM** | b-wave amplitude |  | 285.9 | (249.3, 293.0) |  | 289.9 | (206.5, 367.5) | 0.502 |
|  | a-wave peak time |  | 40.8 | (39.2, 42.4) |  | 40.0 | (38.8, 40.8) | 0.379 |
|  | b-wave peak time |  | 75.6 | (72.0, 82.0) |  | 79.6 | (76.8, 87.2) | 0.257 |
| **SCOTOPIC STANDARD FLASH** | a-wave amplitude |  | 258.8 | (200.9, 278.1) |  | 243.2 | (225.6, 292.2) | 0.494 |
|  | b-wave amplitude |  | 463.1 | (417.6, 475.2) |  | 458.3 | (353.3, 598.5) | 0.967 |
|  | a-wave peak time |  | 20.0 | (18.4, 20.5) |  | 19.2 | (18.4, 20.0) | 0.461 |
|  | b-wave peak time |  | 43.2 | (41.9, 44.9) |  | 45.6 | (44.4, 47.2) | **0.046** |

**Supplementary Table S4: Comparison of electroretinography (ERG) testing success rates between tested at null region and away from null (AFN) in patients with infantile nystagmus**

The Patient ID starts with ‘A’ stand for a patient with albinism while the ID starts with ‘N’ means the patient with IIN. The left gaze showed a negative degree, and the right gaze is showed a positive degree. S.F.: standard flash; AFN: away from null.

##
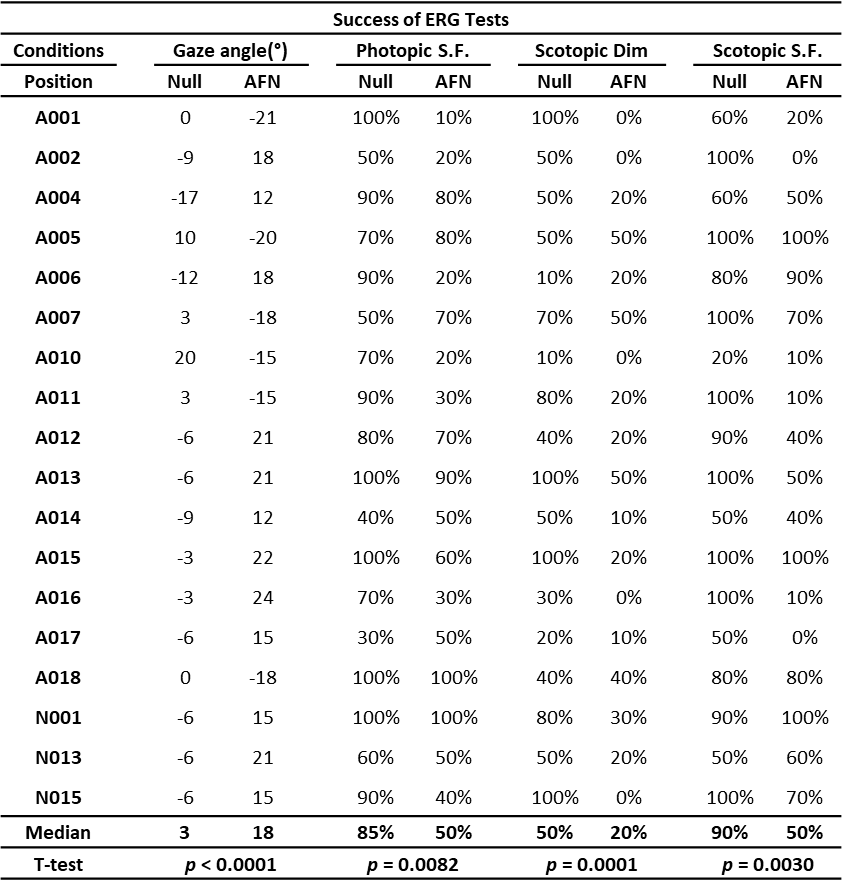


**Supplementary Table S5: Comparisons of electroretinography (ERG) findings between the current study and previous studies.**


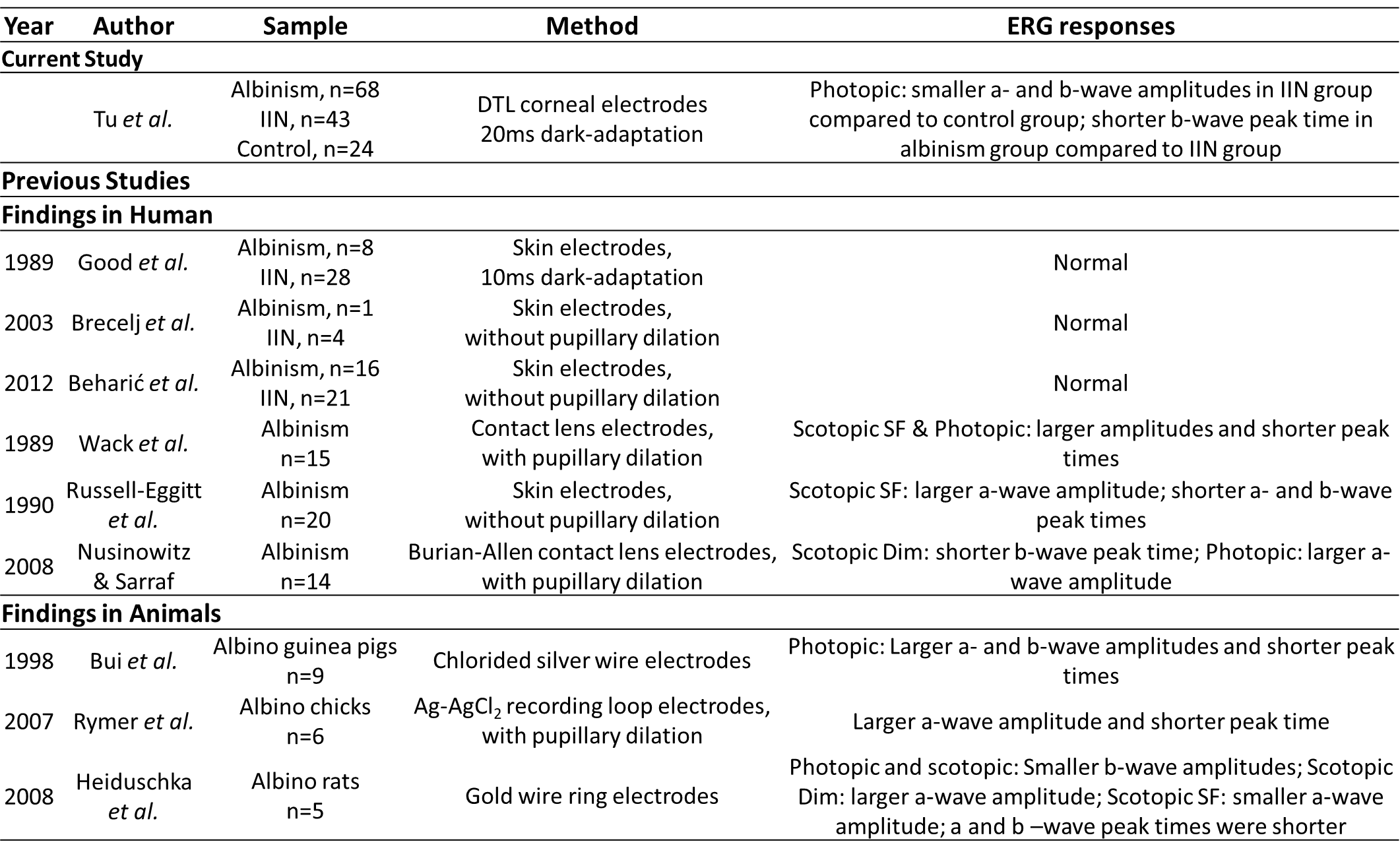


**Reference**

1. Kruijt CC, de Wit GC, Bergen AA, Florijn RJ, Schalij-Delfos NE, van Genderen MM. The Phenotypic Spectrum of Albinism. Ophthalmology. 2018 Dec;125(12):1953-1960. doi: 10.1016/j.ophtha.2018.08.003. Epub 2018 Aug 8. PMID: 30098354.
